# Outcomes of Eltrombopag Treatment and Development of Iron Deficiency in Children with Immune Thrombocytopenia in Turkey

**DOI:** 10.4274/tjh.galenos.2020.2019.0380

**Published:** 2020-08-28

**Authors:** Ayça Koca Yozgat, Göksel Leblebisatan, Sinan Akbayram, Simge Çınar Özel, Zeynep Karakaş, Erol Erduran, Şebnem Yılmaz, Ülker Koçak, Şule Ünal, Gül Nihal Özdemir, Meryem Albayrak, Emine Zengin, Yeşim Oymak, Özcan Bör, Hasan Fatih Çakmaklı, Murat Söker, Dilek Gürlek Gökçebay, Hüseyin Tokgöz, Barış Malbora, Serap Karaman, Tiraje Celkan, İlgen Şaşmaz, Neşe Yaralı, Hale Ören, Ayşegül Ünüvar, Namık Yaşar Özbek

**Affiliations:** 1 Ankara City Hospital, Clinic of Pediatric Hematology, Ankara, Turkey; 2 Çukurova University Faculty of Medicine, Department of Pediatric Hematology, Adana, Turkey; 3 Gaziantep University Faculty of Medicine, Department of Pediatric Hematology, Gaziantep, Turkey; 4 İstanbul University-Cerrahpaşa Cerrahpaşa Faculty of Medicine, Department of Pediatric Hematology, İstanbul, Turkey; 5 İstanbul University, İstanbul Faculty of Medicine, Department of Pediatric Hematology, İstanbul, Turkey; 6 Karadeniz Technical University Faculty of Medicine, Department of Pediatric Hematology, Trabzon, Turkey; 7 Dokuz Eylül University Faculty of Medicine, Department of Pediatric Hematology, İzmir, Turkey; 8 Gazi University Faculty of Medicine, Department of Pediatric Hematology, Ankara, Turkey; 9 Hacettepe University Faculty of Medicine, Department of Pediatric Hematology, Ankara, Turkey; 10 Kanuni Sultan Süleyman Training and Research Hospital, Clinic of Pediatric Hematology, İstanbul, Turkey; 11 Kırıkkale University Faculty of Medicine, Department of Pediatric Hematology, Kırıkkale, Turkey; 12 Kocaeli University Faculty of Medicine, Department of Pediatric Hematology, Kocaeli, Turkey; 13 Dr. Behçet Uz Children’s Training and Research Hospital, Clinic of Pediatric Hematology, İzmir, Turkey; 14 Eskişehir University Faculty of Medicine, Department of Pediatric Hematology, Eskişehir, Turkey; 15 Ankara University Faculty of Medicine, Department of Pediatric Hematology, Ankara, Turkey; 16 Dicle University Faculty of Medicine, Department of Pediatric Hematology, Diyarbakır, Turkey; 17 Ankara Keçiören Training and Research Hospital, Clinic of Pediatric Hematology, Ankara, Turkey; 18 Necmettin Erbakan University Meram Faculty of Medicine, Department of Pediatric Hematology, Konya, Turkey; 19 Yeni Yüzyıl University, Gaziosmanpaşa Hospital, Clinic of Pediatric Hematology, İstanbul, Turkey

**Keywords:** Immune thrombocytopenia, Eltrombopag, Iron deficiency

## Abstract

**Objective:**

Immune thrombocytopenia (ITP) is a rare autoimmune disease and hematologic disorder characterized by reduced platelet counts that can result in significant symptoms, such as bleeding, bruising, epistaxis, or petechiae. The thrombopoietin receptor agonist eltrombopag (EPAG) is a second-line agent used to treat chronic ITP purpura in adults and children.

**Materials and Methods:**

The present retrospective study evaluated the efficacy, safety, and side effects of EPAG treatment in pediatric patients with acute refractory and chronic immune thrombocytopenia, particularly focusing on iron-deficiency anemia.

**Results:**

The diagnosis was chronic ITP in 89 patients and acute refractory ITP in 16 patients. The mean age of patients was 9.5±4.5 years (minimum-maximum: 1.2-18 years) at the beginning of EPAG treatment. The overall response rate was 74.3% (n=78). The mean time for platelet count of ≥50x109/L was 11.6±8 weeks (range: 1-34 weeks). The treatment was stopped for 27 patients (25.7%) at an average of 6.8±9 months (range: 1-38 months). The reason for discontinuation was lack of response in 18 patients, nonadherence in 4 patients, and hepatotoxicity in 2 patients. Response to treatment continued for an average of 4 months after cessation of EPAG in 3 patients.

**Conclusion:**

Results of the current study imply that EPAG is an effective therapeutic option in pediatric patients with acute refractory and chronic ITP. However, patients must be closely monitored for response and side effects during treatment, and especially for iron deficiency.

## Abstract (tr)

**Amaç:**

İmmün trombositopeni (İTP) nadir otoimmün bir hastalık olup, platelet sayısındaki azalmaya bağlı olarak peteşi, ekimoz, epistaksis gibi kanama semptomları ile karakterizedir. Trombopoietin reseptör agonisti eltrombopag (EPAG), yetişkinlerde ve çocuklarda kronik immün trombositopeni tedavisinde kullanılan ikinci basamak bir ajandır.

**Gereç ve Yöntemler:**

Bu retrospektif çalışma, akut refrakter ve kronik immün trombositopenili çocuk hastalarda EPAG tedavisinin etkinliğini, güvenilirliğini, yan etkilerini özellikle de demir eksikliği anemisi gelişimini değerlendirmiştir.

**Bulgular:**

Hastalarımızın 89’u kronik İTP, 16’sı ise akut refrakter İTP tanısı ile takip edilmekte idi. Tedavi başlangıcı sırasında hastalarımızın yaş ortalamaları 9,5±4,5 yıl (1,2-18 yıl) idi. Toplam yanıt oranımız %74,3 (n=78) idi. Platelet sayısının ≥50x109/L olması için geçen ortalama süre 11,6±8 hafta (1-34 hafta) idi. Tedavi 27 hastamızda (%25,7) ortalama 6,8±9 ayda (1-38 ay) kesildi. Tedavi kesim nedenlerimiz; 18 hastada yanıtsızlık, 4 hastada tedaviye uyumsuzluk, 2 hastamızda ise hepatotoksisite idi. Üç hastada ise EPAG tedavisi kesilmesinden sonra yanıt ortalama 4 ay devam etti.

**Sonuç:**

Çalışmamız, EPAG tedavisinin akut refrakter ve kronik İTP’li pediyatrik hastalarda etkili bir terapötik seçenek olduğunu göstermektedir. Bununla birlikte, hastalar EPAG tedavisi sırasında yanıt ve yan etkiler ve özellikle demir eksikliği açısından yakından izlenmelidir.

## Introduction

In most children, immune thrombocytopenia (ITP) is a benign disorder recovering spontaneously within 1 year. Approximately 10%-20% of these patients develop chronic ITP (Ch-ITP), defined as platelet (Plt) count of <100x10^9^/L continuing beyond 1 year in the absence of other causes or disorders [1]. The pathogenesis of Ch-ITP includes autoantibodies directed against Plt glycoproteins leading to Plt destruction as well as impaired megakaryocyte production and maturation [2,3]. Recently, F-cγ polymorphisms have been linked to susceptibility to ITP and progression to chronic disease in children [4].

First-line treatments for children with ITP include corticosteroids, intravenous immunoglobulin, and anti-D immunoglobulin, whereas second-line treatment options are rituximab, splenectomy, and more recently the thrombopoietin receptor agonists (TPO-RA) eltrombopag (EPAG) and romiplostim [5]. EPAG, an oral nonpeptide TPO-RA, binds to a transmembrane site of the thrombopoietin receptor. This binding results in signaling through the JAK/STAT, AKT, and MAPK pathways and facilitates proliferation and differentiation of megakaryocytes that ultimately increase Plt production [6]. The drug has been approved in many countries for use in pediatric patients with Ch-ITP [7,8,9]. Some of the main side effects of EPAG include hepatotoxicity, myelofibrosis, and cataracts. Recently, mobilization of iron after the use of EPAG in patients with aplastic anemia and the development of iron deficiency (ID) in a few children with Ch-ITP have been reported [10,11]. TPO-RA have also been suggested as a first-line treatment choice in patients with refractory ITP (r-ITP) [9].

In this study we aimed to review the effects and side effects, particularly ID/ID anemia (IDA), after the use of EPAG in patients with Ch-ITP.

## Materials and Methods

This retrospective study included children with chronic or acute r-ITP who received EPAG treatment between January 2016 and December 2018. In order to obtain the numbers and data of children who received EPAG treatment, we sent a questionnaire to all pediatric hematology oncology centers (PHOCs) via the Turkish Pediatric Hematology Society. Data were collected from centers that agreed to participate in the study. Ch-ITP and r-ITP were diagnosed as described elsewhere [1,8]. The questionnaire contained data including age, diagnosis, duration of disease, concomitant disorders, bone marrow aspiration and biopsy results, previous treatment(s) and treatment duration, splenectomy status, and eye examination results. Treatment data included the indication for starting EPAG treatment, initial dose of the drug and dose changes, adherence of patients and schedule of drug administration, duration of treatment, indication and time to stop treatment, concurrent treatment(s), drug compliance, the lowest and highest Plt counts before and after treatment, time for Plt count of >50x10^9^/L, and development of ID/IDA during treatment. Patients’ data for hepato/splenomegaly, autoimmunity (ANA, anti-DNA, etc.), viral markers, immunoglobulin profile, bone marrow aspiration, and megakaryocyte morphology and sufficiency at diagnosis were also noted. Follow-up times and characteristics of treatment (side effects and physical and laboratory examinations) were noted for each patient. Eye examinations were performed just before starting the EPAG treatment.

A successful Plt response to therapy was defined as Plt count of ≥50x10^9^/L within 2-4 weeks of treatment and this threshold was determined as the primary treatment goal. We defined ID and anemia on the basis of the criteria of the World Health Organization. Serum ferritin of <12 µg/L in children <5 years of age and of <15 µg/L in children >5 years of age were accepted as ID. If the hemoglobin level was below normal together with ID, the patient was diagnosed with IDA [12].

The study was approved by the ethics committee. Informed consent was received from all parents or caregivers.

### Statistical Analysis

Data were analyzed using SPSS 15.0 (SPSS Inc., Chicago, IL, USA). The normal distribution of continuous variables was evaluated with the Kolmogorov-Smirnov test. Parametric tests were used for variables that were distributed normally, while nonparametric tests were utilized for variables without normal distribution. The correlation between variables was tested with Spearman’s correlation test. Categorical variables were compared with the chi-square test and two independent groups were compared using t-tests and Mann-Whitney U tests. Quantitative variables are demonstrated as mean±standard deviation or median and interquartile range. The confidence interval was 95% and differences associated with a p-value of less than 0.05 were considered as statistically significant.

## Results

### Patient Data

The data of 105 patients from local physicians of 19 PHOCs throughout Turkey were collected (Table 1). The mean age of patients was 6.7±4.3 years (range: 1-16 years, M/F: 56/49) at diagnosis and 9.5±4.5 years (1.2-18 years) at the beginning of EPAG treatment. The diagnosis was Ch-ITP for 89 patients (mean age: 11.1±4.2 [3-18 years; M/F: 48/41]) and acute r-ITP for 16 patients (mean age: 8.4±5.9 [1-18 years; M/F: 8/8]). Treatment was respectively started at the age of 13 and 14 months for 2 patients with r-ITP. The median duration of disease before EPAG therapy was 28 months (range: 12-148 months) for Ch-ITP patients and 11 months (range: 5-24 months) for r-ITP patients. Bone marrow examination was done for all patients before EPAG treatment and revealed results compatible with ITP. Eltrombopag was used as a second-line treatment for all acute patients after the failure of first-line therapies. First-line treatments included intravenous immunoglobulin for 103 patients, steroids for 101 patients, and anti-Rh Ig for 19 patients. Out of 105 patients, 19 patients received three, 80 patients received two, and 6 patients received one of these drugs as first-line treatments. Second-line treatments included mycophenolate mofetil for 14 patients, cyclosporine for 12 patients, and rituximab for 25 patients. The most common indication for EPAG treatment was the lack of response to the first-line treatments (94 patients; 89.5%). Frequent bleeding (56 patients; 53.3%) and the patient’s will to continue daily activities (41 patients; 39%) were other common reasons to start EPAG treatment.

### Laboratory Results

Median Plt count at diagnosis was 8x10^9^/L (range: 2x10^9^/L to 86x10^9^/L). At the beginning of EPAG therapy, it was 10.4x10^9^/L (n=105; range: 1-82). During follow-up, the median Plt counts were 30.5x10^9^/L (n=105, range: 2-371) at the 1^st^ month, 64x10^9^/L (n=60, range: 2-440) at the 6^th^ month, 80x10^9^/L at the 1^st^ year (n=35, range: 8-500), and 98x10^9^/L (n=10, range: 30-1192) at the 2^nd^ year of treatment (Table 2). The mean duration of treatment was 12.3±10 months (range: 1-43 months). Adherence to treatment was good in all but 8 patients (92.4%). Response to EPAG treatment as defined by Plt count of ≥50x10^9^/L was 74.3% (n=78). The mean time for Plt count of ≥50x10^9^/L was 11.6±8 weeks (range: 1-34 weeks). The initial dose of EPAG was 25-50 mg/day according to the patient’s weight and increased to a maximum of 75 mg/day in the event of insufficient response and good tolerance. Dose modification was performed for 67 patients (63.8%). Out of those 67 patients, the dose was decreased or the treatment was discontinued due to thrombocytosis in 12 (11.4%) patients. The treatment was stopped for 27 patients (25.7%) at an average of 6.8±9 months (range: 1-38 months). The reason for discontinuation was lack of response in 18 patients, nonadherence in 4 patients, and hepatotoxicity in 2 patients. Response to treatment continued for an average of 4 months after cessation of EPAG in 3 patients. Bleeding symptoms such as petechiae, ecchymosis, epistaxis, and gingival bleeding were found to be decreased in those who responded to EPAG treatment; hence, they were regarded as having been successfully treated with EPAG. Cataracts and/or thrombosis were not observed in any of our patients during treatment.

### Iron-Deficiency Anemia

According to the laboratory results, ID (n=5) or IDA (n=24) developed at an average of 5.7±3.7 months (range: 1-12 months) in 29 patients (27.6%). Since ID/IDA during the course of EPAG treatment has only been reported very recently, ferritin levels at the beginning of the treatment were not available for most of the patients. Among 26 patients who developed IDA, median hemoglobin levels decreased from 12.6 g/dL (range: 9.8-15.1 g/dL) to 10.7 g/dL (range: 7.1-12.3 g/dL), while median mean corpuscular volume (MCV) decreased from 79.1 fL (range: 71.4-84 fL) to 71 fL (range: 62.3-77.4 fL) and median red blood cell distribution width (RDW) increased from 14 (range: 13-19.8) to 16.5 (range: 15-23.1) during treatment. There was a statistically significant difference between the hemoglobin, MCV, and RDW values of our patients with and without ID (p<0.05). Among the 29 patients with ID/IDA, data concerning ferritin levels at the beginning of EPAG treatment were available for 12 patients. Median ferritin levels were 24 ng/mL (range: 18-56 ng/mL) in these 12 patients at the beginning of EPAG treatment and 7.3 ng/mL (range: 4.4-13 ng/mL) when ID/IDA developed. The median ferritin levels in the 29 patients who developed ID/IDA were found to be decreased to 8.6 ng/mL (range: 4.4-14 ng/mL) during EPAG treatment. Of the 26 patients who developed IDA, only 2 of them had low initial hemoglobin and MCV values, but ferritin values of these 2 patients were not available, and the hemoglobin and MCV values of the other patients who developed IDA were within normal limits at the beginning of EPAG treatment. The Plt counts of the 29 ID/IDA patients were higher compared to those of iron-competent patients at the 1^st ^and 6^th^ months of EPAG treatment. Although statistically not significant, median Plt counts were 31.5x10^9^/L (range: 3x10^9^/L to 118x10^9^/L) at the 1^st^ month and 65.2x10^9^/L (range: 8x10^9^/L to 440x10^9^/L) at the 6^th^ month in patients who developed ID/IDA, whereas these values were 29x10^9^/L (range: 2x10^9^/L to 371x10^9^/L) at the 1^st^ month and 56.8x10^9^/L (range: 3x10^9^/L to 336x10^9^/L) at the 6^th^ month in iron-competent patients (p>0.05). We found no statistical relationship between EPAG dose and development of ID/IDA. All patients who developed ID/IDA responded to oral iron treatment.

## Discussion

Pediatric Ch-ITP is a potentially serious bleeding disorder that may also impact the child’s quality of life due to restriction of physical or social activities [13]. In Ch-ITP patients who fail to respond or who relapse following first-line treatments, second-line treatments are usually needed. The TPO-RA EPAG and romiplostim are among second-line treatments that not only provide a Plt response in the majority of the patients (80%) but also maintain that response throughout the treatment course [14,15]. Eltrombopag is an oral, nonpeptide thrombopoietin receptor agonist approved for both adult and pediatric patients with Ch-ITP. The efficacy and safety of EPAG in pediatric patients has been demonstrated in 2 international, randomized, double-blind, placebo-controlled trials, PETIT1 and PETIT2. The results of these studies were consistent and demonstrated that EPAG improves Plt counts, helps children reduce or discontinue concomitant ITP medications, and decreases the need for rescue therapy. The results revealed that at least in 40% of the patients, there was a significant rise of Plt levels with the use of EPAG [16,17]. The most common adverse events related to EPAG treatment were elevation of the liver enzymes, which was generally mild, without signs of impaired liver function. Our study showed similar results concerning the efficacy and durability of response. In our study, treatment was discontinued for only two patients (2%) due to elevation of liver enzymes. We did not observe any thrombosis or cataracts; however, the number of patients and the duration of follow-up were not sufficient to draw a conclusion in this regard.

In recent preclinical studies, EPAG has been shown to chelate intracellular iron in malignant cells independently of thrombopoietin receptors. In addition, the inhibition of cell division and differentiation by blocking malignant cells in the G1 phase has been observed. As a result, survival was improved in mouse leukemia models [18,19,20]. Punzo et al. [21] also showed that EPAG binds to metal ions and especially iron(III). They confirmed that EPAG is a powerful iron chelator, and when it was used with deferasirox, the iron chelation capacity increased in thalassemia patients [21]. In another study, EPAG therapy was shown to inhibit replication of human cytomegalovirus infection by iron chelation [22]. Following these findings, a clinical study with a limited number of children with Ch-ITP revealed that EPAG treatment reduced the patients’ MCV and ferritin levels, ultimately causing IDA [23]. The results of our study were similar to those of these previous studies. We observed ID/IDA in about a quarter of the patients treated with EPAG. Our study suggests that hemoglobin, erythrocyte indices, and ferritin levels should be monitored in patients treated with EPAG. Response to iron treatment was good, implying that early diagnosis of ID is important to start iron treatment in order to prevent IDA. Long-term follow-up is mandatory to show whether ID/IDA is recurrent in patients continuing on EPAG treatment. IDA is a well-known cause of secondary thrombocytosis, usually of mild to moderate degree [24]. Although the mechanism of thrombocytosis is not completely understood, accelerated megakaryopoiesis due to elevated megakaryocytic growth factors such as thrombopoietin, interleukin (IL)-6, or IL-11 has been assumed [25]. In our study, we observed that the Plt count was higher in patients who developed ID/IDA at the 1^st^ and 6^th^ months. Although the results were not statistically significant, the treatment response was better in patients who had ID/IDA compared to that of iron-competent patients. This finding needs further studies to clarify the underlying mechanism of how ID during EPAG treatment causes thrombocytosis.

## Conclusion

Our multicenter study provides evidence that EPAG is an effective and safe agent in the treatment of children with chronic or acute refractory ITP. Treatment with EPAG is associated with hepatotoxicity in some patients. The most important finding was ID/IDA during treatment, which necessitates careful evaluation of patients during treatment. A larger prospective study could address these concerns more definitively.

## Figures and Tables

**Table 1:**
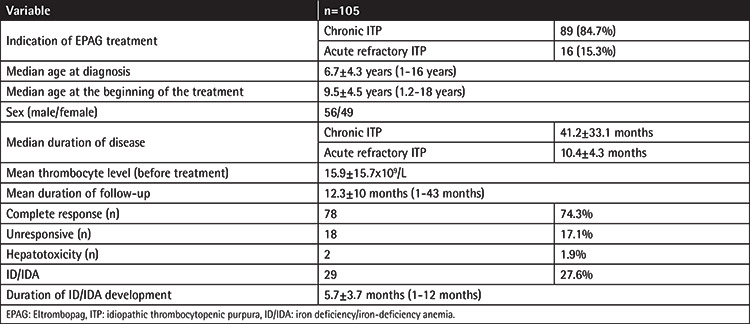
Descriptive and clinical data of patients with ITP.

**Table 2:**
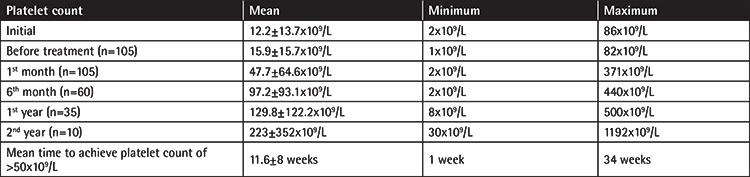
Data related to platelet count during the course of eltrombopag treatment.
